# MicroRNA regulation and dysregulation in epilepsy

**DOI:** 10.3389/fncel.2013.00172

**Published:** 2013-10-04

**Authors:** Danyella B. Dogini, Simoni H. Avansini, Andre S. Vieira, Iscia Lopes-Cendes

**Affiliations:** Department of Medical Genetics, School of Medical Sciences, University of Campinas, CampinasSão Paulo, Brazil

**Keywords:** microRNAs, epilepsy, temporal lobe, cortical malformations, animal models

## Abstract

Epilepsy, one of the most frequent neurological disorders, represents a group of diseases that have in common the clinical occurrence of seizures. The pathogenesis of different types of epilepsy involves many important biological pathways; some of which have been shown to be regulated by microRNAs (miRNAs). In this paper, we will critically review relevant studies regarding the role of miRNAs in epilepsy. Overall, the most common type of epilepsy in the adult population is temporal lobe epilepsy (TLE), and the form associated with mesial temporal sclerosis (MTS), called mesial TLE, is particularly relevant due to the high frequency of resistance to clinical treatment. There are several target studies, as well few genome-wide miRNA expression profiling studies reporting abnormal miRNA expression in tissue with MTS, both in patients and in animal models. Overall, these studies show a fine correlation between miRNA regulation/dysregulation and inflammation, seizure-induced neuronal death and other relevant biological pathways. Furthermore, expression of many miRNAs is dynamically regulated during neurogenesis and its dysregulation may play a role in the process of cerebral corticogenesis leading to malformations of cortical development (MCD), which represent one of the major causes of drug-resistant epilepsy. In addition, there are reports of miRNAs involved in cell proliferation, fate specification, and neuronal maturation and these processes are tightly linked to the pathogenesis of MCD. Large-scale analyzes of miRNA expression in animal models with induced status epilepticus have demonstrated changes in a selected group of miRNAs thought to be involved in the regulation of cell death, synaptic reorganization, neuroinflammation, and neural excitability. In addition, knocking-down specific miRNAs in these animals have demonstrated that this may consist in a promising therapeutic intervention.

## MicroRNAs IN HUMAN MESIAL TEMPORAL LOBE EPILEPSY

Epileptic seizures are the clinical manifestations that reflect a temporary dysfunction of a set of neurons in the brain (Engel, 2001). Epilepsy has a high prevalence in the population, about 1.5–2% and it is considered a public health problem since it has important social and economic impact (Annegers et al., 1996; Borges et al., 2004). Because of its high prevalence and severity, temporal lobe epilepsy (TLE) is one of the most studied types of epilepsy. In TLE complete seizure control with drug treatment is achieved in less than 50% of patients (Sander, 1993; Mattson, 1994). The most common form of TLE is mesial TLE (MTLE), which has the symptoms generated by the involvement of the medial temporal lobe structures (Engel, 2001). Resistance to drug treatment is a crucial problem for patients with MTLE and surgery to remove the affected brain area is, in many cases, a successful therapeutic strategy (Engel, 2001). Surgical specimens in MTLE most frequently show mesial temporal sclerosis (MTS), which is a pathological condition with specific features, including selective neural loss and gliosis in the CA1 hippocampal region (Wieser, 2004). Other changes may include dispersion of the granule cells in the *dentate gyrus*, neurogenesis of granule cell and synaptic reorganization of the mossy fibers (Thom, 2004). Focal lesions and malformations of cortical development (MCD; cortical dysplasia) may represent other findings in patients with drug refractory MTLE (Blumcke et al., 2002; Thom, 2004).

It has been demonstrated that different microRNAs (miRNAs) may have different expression pattern in different brain regions, and these differences in distribution may be related to the preferential concentration of synaptically localized mRNA targeted by these miRNAs (Pichardo-Casas et al., 2012). Furthermore, these differences in concentration could be modulated by epileptogenic activity (Pichardo-Casas et al., 2012). McKiernan et al. (2012a) detected a significant expression of about 200 miRNAs in healthy human hippocampus. However, when working with tissue obtained from patients with MTLE and using TaqMan® low-density arrays (TLDAs) they found a large-scale reduction of miRNA expression, with 51% of miRNAs tested expressed at lower levels than in controls and about 24% not detectable in epileptic tissue. In addition, these authors showed that a possible mechanism involved in failure of mature miRNA expression was a significant decreased expression of DICER, an enzyme required for the generation of mature miRNAs (McKiernan et al., 2012a).

MicroRNA may also have a significant role in inflammation pathways which have been shown to be involved in MTLE (Vezzani et al., 2013). MiR-146a is significantly up-regulated in tissue obtained from patients with MTLE (Aronica et al., 2010; Omran et al., 2012). MiR-146a has been implicated in regulation of astrocyte-mediated inflammatory response (Iyer et al., 2012). In addition, *in vitro* experiments showed a significant up-regulation of miR-146a in astrocytes when exposed to interleukin-1 beta (IL-1b) stimulation, which is known to be up-regulated in the acute phase of some animal models of MTLE (Aronica and Crino, 2011). Another miRNA that has been associated with inflammatory pathways in MTLE is miR-155 (Ashhab et al., 2013). It has been demonstrated an increase in the expression of miR-155 in hippocampal tissue from children with MTLE, as well as in an immature rat epilepsy model. Moreover, the observed increase in miR-155 expression correlates with an increase in TNF-α in the nervous tissue (Ashhab et al., 2013).

It is well known that neuronal death related to seizures involves direct glutamate-driven excitotoxic necrosis. MiR-34a, which belongs to a conserved miRNA family, appears to have a direct pro-apoptotic effect in cells and regulates p53 (Hermeking, 2010). In addition, up-regulation or overexpression of this miR-34a promotes apoptosis in a variety of non-neuronal cell (Chang et al., 2007). Therefore, it has been suggested recently, that miR-34a could represent a key player in the mechanism underlying neuronal death induced by seizures (Hu et al., 2012; Sano et al., 2012).

MicroRNAs may also be involved in enzyme-related epileptic pathology. It is known that adenosine is an endogenous regulator of hippocampal activity and that it has a potent anti-ictogenic and neuroprotective properties (Bjorklund et al., 2008), as well as it is crucial for astrocyte physiology (Boison, 2009). Synaptic levels of adenosine in adult brain are largely regulated by an astrocyte-based adenosine-cycle (Boison, 2009). Adenosine is rapidly phosphorylated by adenosine kinase (ADK), which is almost exclusively expressed in astrocytes (Studer et al., 2006). According to the ADK hypothesis of epileptogenesis (Boison, 2009), any type of brain injury can produce astrogliosis, which leads to the up-regulation of ADK, creating focal adenosine deficiency as a direct cause of seizures. Using lentiviral vectors in human mesenchymal stem cells coexpressing miRNA against ADK transduction, Ren and Boison (2010) found about 80% of ADK down-regulation. These results suggest that miRNAs are important regulators of seizure-induced neuronal death and that these molecules might be used as novel therapeutic targets in the treatment of epilepsy. Some other miRNAs, such as miR-124, miR-134, miR-132, miR-196b (You et al., 2012; Peng et al., 2013) have also been reported to be involved in epilepsy (**Table 1**).

**Table 1:** MicroRNAs potentially involved in epilepsy.

MicroRNA	Human studies/experimental models	Potential role in epilepsy	Reference
miR-124	Human; immature rat	Potential role in mesial temporal lobe epilepsy; control cell proliferation	Makeyev et al. (2007); Peng et al. (2013)
miR-132	Human; mouse kainic acid	Associated to neuronal activation and synaptic plasticity	Vo et al. (2005)
miR-134	Human (*in vitro* experiments); mouse kainic acid	Suppresses evoked seizures; regulates cell migration	Jimenez-Mateos et al. (2012)
miR-137	Human; rat	Regulates cell proliferation; critical for neural differentiation	Krichevsky et al. (2006); Smrt et al. (2010).
miR-146	Human; mouse; rat	Regulation of astrocyte-mediated inflammatory response; neural inflammation	Lukiw et al. (2008); Nakasa et al. (2008), Pauley et al. (2008); Sonkoly et al. (2008), Aronica and Crino (2011); Iyer et al. (2012), Cheng et al. (2013)
miR-153, miR-324, miR-181a	Human; rat	Critical role in neural differentiation	Smrt et al. (2010); Agostini et al. (2011), Stappert et al. (2013)
miR-184	Human; mouse kainic acid	Regulates cell proliferation; neuroprotective effect	Krichevsky et al. (2006); Makeyev et al. (2007), Silber et al. (2008); Liu et al. (2010a), Zhao et al. (2010); McKiernan et al. (2012b)
miR-196b	Human	Associated with the occurrence of seizures	You et al. (2012)
miR-21	Rat pilocarpine	Possible associated with increased neuronal loss following *status epilepticus*	Risbud and Porter (2013)
miR-34a	Human; rat pilocarpine; mouse kainic acid	Involved in seizure-induced neuronal death; critical for neural differentiation	Agostini et al. (2011); Hu et al. (2012), Sano et al. (2012)
miR-9	Human (*in vitro* experiments)	Regulates cell proliferation; promotes cell migration; accelerates neural differentiation	Krichevsky et al. (2006); Delaloy et al. (2010)
let-7b	Human; rat kainic acid	Regulates cell proliferation	Krichevsky et al. (2006); Makeyev et al. (2007), Silber et al. (2008); Liu et al. (2010b), Zhao et al. (2010)

## MicroRNAs AND MALFORMATIONS OF CORTICAL DEVELOPMENT

Malformations of cortical development are a frequent cause of medically intractable epilepsy. It has been estimated that 25–40% of drug-resistant epilepsies are caused by MCD (Guerrini et al., 2003). The development of the human cerebral cortex is a dynamic and complex process. These processes are orchestrated by interactions between extracellular and intracellular signaling cues and any disruption of these cellular processes can result in cortical malformations (Sisodiya, 2004; Guillemot et al., 2006; Guerrini et al., 2008; McLoughlin et al., 2012).

**FIGURE 1:**
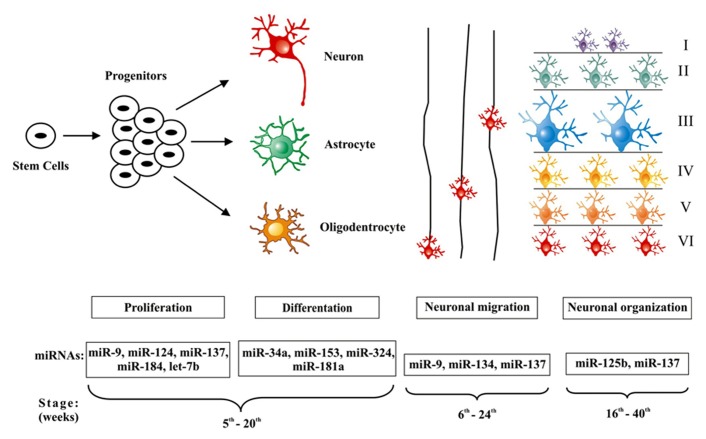
**MicroRNAs involved in the regulation of cerebral cortex development.** The figure demonstrates microRNAs that have been associated with the three main phases of cortical development. In the first stage, stem cells generate progenitors that are not yet committed to differentiation and can produce neurons, astrocytes, and oligodendrocytes; the concomitant steps of proliferation and differentiation (5th– 20th weeks of gestation) are regulated by a set of microRNAs: miR-9, miR-124, miR-137, miR-184, let-7b and miR-34a, miR-153, miR-324, miR- 181a. Successive waves of neurons migrate (6th–24th weeks of gestation) from the ventricular regions, along radial glial cells, toward the more external areas of the cortex, these processes are regulated by miR-9, miR-134, and miR-137. Finally, the organization of cortical layers (16th–40th, weeks of gestation) is regulated at this stage through miR-137 and miR-125b.

Molecular biology and genetic studies have greatly expanded knowledge on cortical neurogenesis so that several disorders of cortical development have been recognized and, for some of them, specific causative genetic defects have been identified (Aronica et al., 2012). Furthermore, recent data support a major role for miRNAs in fine-tuning of signaling pathways that control the concomitant phases of corticogenesis. Supporting this notion, we have previously shown that groups of miRNAs are differentially regulated during normal mouse brain development (Dogini et al., 2008). Small alterations of their expression have been associated with a variety of neurological disorders (Volvert et al., 2012). Nevertheless, few studies have investigated the possible role of miRNAs in the pathogenesis and/or epileptogenesis of MCDs. Therefore, we aim in the next few paragraphs to summarize current knowledge about miRNAs and cerebral corticogenesis (**Figure 1**) and how its dysregulation may play a role in the process leading to MCDs and ultimately to epileptogenesis as seen in some of these lesions (**Table 1**).

### MicroRNAs IN NEURONAL AND GLIAL PROLIFERATION AND DIFFERENTIATION

The first step of cortical development is cellular proliferation and differentiation, which takes place between the 5th week and 20th week of gestation (Sidman and Rakic, 1973; Guerrini and Barba, 2010). Microcephaly, tuberous sclerosis, and focal cortical dysplasia (FCD) have been considered to be malformations of these phases. MiR-9, miR-124, miR-137, miR-184, and let-7b were shown to control cell proliferation in the cortex (Krichevsky et al., 2006; Makeyev et al., 2007; Silber et al., 2008; Liu et al., 2010a; Zhao et al., 2010). In addition, loss of miR-9 expression, a brain-specific miRNA, suppresses the proliferation and promotes the migration of human embryonic neural progenitors, cultured *in vitro*, by targeting stathmin, which increases microtubule instability in migrating neuroblasts (Delaloy et al., 2010). In the mouse embryonic brain, miR-9 suppressed TLX expression, resulting in a reduction of neural stem cell proliferation and an acceleration of neural differentiation (Zhao et al., 2009).

The cellular complexity of the cerebral cortex emerges through specification of cortical progenitors into distinct subtypes of neurons and glia that reach cortical layers (Kriegstein and Alvarez-Buylla, 2009). Changes in gene expression underlie the transition from progenitors to neurons (Guillemot et al., 2006). Conditional removal of Dicer in the cortex affects this process. Kawase-Koga et al. (2009) reported that the cerebral cortex of deficient Dicer-mice showed a significant reduction in cortical thickness, caused by a reduction in neural stem cells and neural progenitors with increased apoptosis and impaired neuronal differentiation. In the same way, it has been observed an inability to generate both neurons and glial cells in the embryonic cerebral cortex of a Dicer-null mouse, and that this enzyme plays a role in maintaining the phenotype of neural stem cells during neuronal differentiation (Andersson et al., 2010). Other miRNAs have also been reported as critical for neural differentiation. These include miR-137, miR34a, miR-153, miR-324, and miR-181a (Smrt et al., 2010; Agostini et al., 2011; Stappert et al., 2013).

Focal cortical dysplasia is characterized by a spectrum of abnormalities in the development of the laminar structure of the human cerebral cortex. Microscopically, FCD is usually associated with cell abnormalities, giant/dysmorphic neurons and balloon cells (Palmini et al., 2004; Guerrini et al., 2008; Sisodiya et al., 2009; Blumcke et al., 2011). As FCDs are the most frequent epileptogenic malformation, susceptible to surgical treatment, it is of great importance to understand the mechanisms underlying epileptogenesis in FCDs (Aronica et al., 2012; Hauptman and Mathern, 2012; Sakakibara et al., 2012). In this context, Iyer et al. (2012) evaluated function of miR-146a in response to pro-inflammatory stimuli and found, by using *in situ* hybridization, increased expression of miR-146a in reactive astrocytes which are abundantly present within the dysplastic cortex in FCD IIb. This observation suggests a role for miR-146a in an astrocyte-mediated mechanism predisposing to seizure in FCDs.

### MicroRNAs IN NEURONAL MIGRATION

In humans, neuronal migration occurs from 6th–7th weeks till approximately 20th–24th weeks of gestation (Sidman and Rakic, 1973; Guerrini and Barba, 2010). Abnormalities disrupting neuronal migration result in highly epileptogenic lesions, causing severe neurological impairment, such as those found in periventricular nodular heterotopia, subcortical heterotopias, and lissencephaly (Guerrini and Parrini, 2010). Doublecortin (Dcx) regulates tangential and radial neuron migration and has been implicated in the pathogenesis of lissencephaly and subcortical heterotopias (Reiner et al., 2006). Gaughwin et al. (2011) demonstrate that miR-134 regulates cell migration *in vitro* and down-regulates Dcx protein *in vivo*, thereby attenuating neuronal migration.

Experiments using neural stem cells of embryonic mouse brains suggest that miR-137 triggered premature differentiation and outward migration through regulation of a lysine-specific histone demethylase (LSD1; Sun et al., 2011). Moreover, the transfection of exogenous miR-125b increased migration of neural stem/progenitor cells compared to a control group (Cui et al., 2012).

A mice model constructed with Dicer depletion, by the Nestin-Cre system revealed a critical role for Dicer in cortical migration (McLoughlin et al., 2012). There was a sevenfold increase in Dcx expression that may have contributed to the premature maturation of neurons in inappropriate regions, which in turn may led to complete cortical disorganization (McLoughlin et al., 2012). Shibata et al. (2011) observed, after reduction of miR-9 expression, that cortical layers were reduced and that the tangential migration of interneurons from basal forebrain was impaired.

### MicroRNAs IN NEURONAL ORGANIZATION

The third stage in cortical development is cortical organization. When migration is complete, the cortex is a six-layered structure, with each layer containing different types of neurons (Guerrini and Barba, 2010). Polymicrogyria and schizencephaly have been considered to be malformations of this post-migrational cortical organization stage. Two miRNAs have been shown to regulate key processes at this stage. MiR-137 which regulates neuronal maturation by inhibiting dendrite formation through binding Mind bomb 1 (Mibl; Smrt et al., 2010), and miR-125b which seems to have a similar role, since overexpression of miR-125b leads to longer and thinner dendritic spines (Edbauer et al., 2010).

## MicroRNAs AND ANIMAL MODELS OF EPILEPSY

Induced animal models are one of the most used tools to study the pathophysiology of different types of epilepsy and they have been most frequently used in MTLE. In these models, animals present behavioral, electroencephalographic, and neuropathological features in the limbic structures similar to those observed in patients with MTLE (Avanzini et al., 1993; Lothman et al., 1995; Engel, 1996).

One of the first miRNAs shown to be differentially expressed in the hippocampus in an induced animal model was miR-132 (Nudelman et al., 2010). These authors observed an increase in the expression of miR-132 in the hippocampus 8 h after the administration of the convulsant drug pilocarpine in mice. In neurons, miR-132 expression is induced by electrical activity and the action of neurotrophins, consequently its proposed role would be the regulation of synaptic plasticity-related genes (Vo et al., 2005; Wayman et al., 2008). Another miRNA that was initially explored in epilepsy experimental models was miR-146a (Aronica et al., 2010). This miRNA can be induced by pro-inflammatory cytokines, such as IL-1b, and it is up-regulated in various human disorders associated with inflammatory response (Lukiw et al., 2008; Nakasa et al., 2008; Pauley et al., 2008; Sonkoly et al., 2008; Cheng et al., 2013). In a rat model of MTLE induced by repetitive electrical stimulation of the perforant pathway, it was observed that miR-146a was up-regulated in the CA3 hippocampus subfield 1 week (latent phase) and 3 months (chronic phase) after the episode of *status epilepticus*. In these experiments, the observation by *in situ* hybridization of miR-146 expression in hippocampus reactive astrocytes further indicated a possible role for this miRNA in neural inflammation. However, the exact genes regulated by miR-146 in the hippocampus remains to be determined.

Subsequently, with an increasing interest in the possible role of regulatory RNAs in epilepsy, large-scale analyzes of miRNA expression profile by either hybridization or TaqMan® arrays were undertaken in the hippocampus of animals with induced epilepsy (Liu et al., 2010b; Jimenez-Mateos et al., 2011; Song et al., 2011; Hu et al., 2012; McKiernan et al., 2012b; Pichardo-Casas et al., 2012; Peng et al., 2013; Risbud and Porter, 2013). Analyzes were performed on the lithium-pilocarpine model (Song et al., 2011; Hu et al., 2012), systemic pilocarpine (Risbud and Porter, 2013), systemic kainic acid (Liu et al., 2010b; McKiernan et al., 2012b; Pichardo-Casas et al., 2012), intra-amygdala kainic acid (Jimenez-Mateos et al., 2011), with time points ranging from a few hours (McKiernan et al., 2012b) to months after *status epilepticus* (Song et al., 2011; Hu et al., 2012). All studies found a significant number of miRNAs differentially regulated in the epileptic state when compared to control animals, indicating a tight regulation of miRNAs associated with the events observed in induced epilepsy models. Some miRNAs were found to be differentially expressed, such as miR-34a (Hu et al., 2012; Sano et al., 2012) or miR-132 (Nudelman et al., 2010; Jimenez-Mateos et al., 2011). However, a coherent interpretation of the results produced by the above mentioned experiments is hindered by the still incomplete knowledge of miRNAs regulated genes in the hippocampus and by the heterogeneity of findings obtained by different studies.

The apparent lack of reproducibility in the miRNA expression profile experiments may be explained by the diversity in animal models, time points, and even hippocampal structures analyzed. Moreover, miRNA expression was profiled employing microarrays (Song et al., 2011; Hu et al., 2012; Pichardo-Casas et al., 2012; Risbud and Porter, 2013) or TLDAs (Liu et al., 2010b; Eacker et al., 2011; Jimenez-Mateos et al., 2011; McKiernan et al., 2012b). As a consequence, differences on the sensibility and specificity of both techniques may be responsible for part of the diversity observed in the published literature. In addition, a critical point to be considered is that some studies analyzed whole hippocampus homogenates (Liu et al., 2010b; Song et al., 2011; Hu et al., 2012; Pichardo-Casas et al., 2012; Peng et al., 2013; Risbud and Porter, 2013) and others were restricted to the CA3 subfield (Jimenez-Mateos et al., 2011; McKiernan et al., 2012b). It is known that the different hippocampus subfields are molecularly diverse (Lein, 2004; Greene et al., 2009). Therefore, analyzes of whole hippocampus homogenates certainly dilutes subfield-specific changes that may take place in these epilepsy models. Strategies such as laser capture microdissection of different hippocampus subfields could circumvent the exposed shortcomings of whole homogenate strategies, improving the ability of an experiment to detect more subtle and spatially restricted changes in miRNA regulation. Furthermore, since different hippocampus subfields have different functional characteristics, sensibility to neurodegeneration and contributions to the establishment of an epileptic state (Becker et al., 2003; Majores et al., 2004), a separate analyzes of miRNA profile in each structure certainly would facilitate data interpretation. Another point to be considered is that the translation of these animal models miRNA expression findings to human MTLE could be hindered by the fact that many patients do not present an initial precipitating event (Van Paesschen et al., 1997). Moreover the occurrence of an episode of *status epilepticus* is uncommon in human MTLE. Such a diversity of models and analyzes strategies present in the literature poses an advantage, since the differentially regulated miRNAs common to all studies may indicate the presence of a common mechanism underlying the epileptogenic process. However, care should be taken when employing rodent data in the effort of understanding human MTLE miRNA associated mechanisms due to the existence of many primate-specific miRNAs (Bentwich et al., 2005). Therefore some mechanisms may only be found with the direct analysis of tissue from patients that undergo epilepsy surgery.

As already noted, many of the functional implications of the identified differentially expressed miRNAs in the hippocampus of animals with induced epilepsy are still unknown. Antagomirs are stable, locked nucleic acids, engineered RNA oligonucleotides that can recognize, based on sequence complementarity, specific miRNAs, inducing its degradation (Krutzfeldt et al., 2005, 2007). These engineered molecules consist in valuable tools for probing miRNAs function *in vivo*, and indeed, functional studies were undertaken in some epilepsy animal models. The induction of low intensity seizures renders animals resistant to subsequent induction of an epileptic state, a phenomena termed epileptic tolerance (for a review see Jimenez-Mateos and Henshall, 2009). It was observed that miR-132 was down-regulated in mice CA3 subfield after seizure preconditioning (Jimenez-Mateos et al., 2011). In the same study, the authors observed that the reduction in expression of miR-132, by the intracerebroventricular administration of an antagomir directed to this miRNA, reduced neuronal loss in the hippocampus after the induction of *status epilepticus* in mice. In the hippocampus miR-132 regulates mRNAs such as acetylcholinesterase or the GTPase activator p250GAP (Hanin and Soreq, 2011; Shaltiel et al., 2013). Furthermore, miR-132 has been previously associated with synaptic plasticity (Vo et al., 2005; Wayman et al., 2008). However, the miR-132 gene targets responsible for the facilitation of neuronal death remain to be determined. Yet another study exploring the role of miRNAs in epileptic tolerance, found an increase in the expression of miR-184 after preconditioning by systemic administration of a low dose of kainic acid (McKiernan et al., 2012b). Subsequently, these authors demonstrated that reduction of miR-184 by intracerebroventricular administration of an antagomir directed to this miRNA reduced the neuroprotective effect of preconditioning on hippocampal neurons, restoring the levels of neuronal death observed when *status epilepticus* was induced without preconditioning. The mRNAs that may interact *in vivo* with miR-184 in the hippocampus are not determined and the mechanism responsible for this miRNA-mediated neuroprotection in the hippocampal CA3 subfield is also unknown. Finally, miR-34a was shown to be up-regulated in different epilepsy animal models and its involvement in neuronal death in the hippocampus was probed with the use of antagomirs (Hu et al., 2012; Sano et al., 2012). The down-regulation of miR-34a by intracerebroventricular injection of antagomirs reduced neuronal death observed in the hippocampus in a lithium-pilocarpine epilepsy model (Hu et al., 2012), but it had no effect on an intra-amygdala kainic acid injection model in mice (Sano et al., 2012). The difference in the experiments outcome may be related to the different models, species and time points analyzed. It is believed that miR-34a may regulate expression of apoptosis-related genes in the hippocampus; however, further experiments are needed to confirm these observations.

Among the functional studies involving miRNAs, the one that explored the role of miR-134 in experimental epilepsy is noteworthy. In an intra-amygdala kainic acid injection epilepsy model in mice, it was observed an increase in the expression level of miR-134 following *status epilepticus*. Furthermore, this miRNA was shown to be expressed by pyramidal neurons in CA3, by interneurons in the hilus and by neocortical as well as amygdala neurons (Jimenez-Mateos et al., 2012). In the same study, the reduction of miR-134 expression by intracerebroventricular injection of antagomirs induced a decrease in CA3 pyramidal neurons spine density and, remarkably, it significantly reduced the severity of the induced seizures following intra-amygdala kainic acid injection. The authors also demonstrated that the induced down-regulation of this single miRNA enhanced resistance to evoked seizures resulting in reduction in all events associated with experimental induction of epilepsy, namely neuronal loss, gliosis, sprouting, and subsequent spontaneous recurrent seizures.

In conclusion, miRNAs are emerging as key regulators of sets of genes involved in the events that take place during epileptogenesis and chronic epilepsy states. Additionally, functional studies employing antagomirs indicate that these regulatory RNAs as promising targets for new possible strategies in the treatment of epilepsy.

## Conflict of Interest Statement

The authors declare that the research was conducted in the absence of any commercial or financial relationships that could be construed as a potential conflict of interest.
